# Variation trend prediction of ground-level ozone concentrations with high-resolution using landscape pattern data

**DOI:** 10.1371/journal.pone.0294038

**Published:** 2023-11-16

**Authors:** Yingying Mei, Xueqi Xiang, Zhenwei Wang, Deping Xiang

**Affiliations:** 1 School of Public Administration, Hubei University, Wuhan, China; 2 Department of Sociology, Zhongnan University of Economics and Law, Wuhan, China; 3 School of Sociology, Huazhong University of Science and Technology, Wuhan, China; UNITEN: Universiti Tenaga Nasional, MALAYSIA

## Abstract

Scientifically configuring landscape patterns based on their relationship with ground-level ozone concentrations (GOCs) is an effective way to prevent and control ground-level ozone pollution. In this paper, a GOC variation trend prediction model (hybrid model) combining a generalized linear model (GLM) and a logistic regression model (LRM) was established to analyze the spatiotemporal variation patterns in GOCs as well as their responses to landscape patterns. The model exhibited satisfactory performance, with percent of samples correctly predicted (*PCP*) value of 82.33% and area under receiver operating characteristics curve (*AUC*) value of 0.70. Using the hybrid model, the per-pixel rise probability of annual average GOCs at a spatial resolution of 1 km in Shenzhen were generated. The results showed that (1) annual average GOCs were increasing in Shenzhen from 2015 to 2020, and had obvious spatial differences, with a higher value in the west and a lower value in the east; (2) variation trend in GOCs was significant positively correlated with landscape heterogeneity (*HET*), while significant negatively correlated with dominance (*DMG*) and contagion (*CON*); (3) GOCs in Shenzhen has a great risk of rising, especially in GuangMing, PingShan, LongGang, LuoHu and BaoAn. The results provide not only a preliminary index for estimating the GOC variation trend in the absence of air quality monitoring data but also guidance for landscape optimizing design from the perspective of controlling ground-level ozone pollution.

## Introduction

Air pollution is closely related to landscape types [1, 2]. With the growth of the human population and intense urbanization, landscape change has been significant during the past several decades [3]. Different landscape types present different atmospheric regulation benefits [4, 5]. The relationship between landscape type and air pollution provides important information for the collaborative promotion of landscape optimization design and air pollution control, which is of great significance for sustainable development.

In China, irresponsible landscape planning during urbanization process has caused serious ground-level ozone pollution in recent years, which is particularly common in developed cities and their surrounding areas [6, 7]. As urbanization has entered a period of rapid growth, the impact of dramatic changes in landscape types and their configuration modes on ground-level ozone concentrations (GOCs) has become prominent, bringing challenges to ground-level ozone pollution control. Ground-level ozone is a secondary pollutant formed by the photochemical reactions of atmospheric precursors such as volatile organic compounds (VOCs) and nitrogen oxides (NOx) [8], and its formation largely depends on the ratio of VOCs/NOx [9]. China has actively promoted reductions in the emissions of ground-level ozone precursors, but ground-level ozone pollution is still serious [10]. High GOCs are extremely harmful to human health, vegetation growth and the ecological environment [11–14]. Social development inevitable leads to urbanization and landscape upheaval. Therefore, it is urgent to explore the effect of landscape types on GOCs and optimize landscape design to alleviate ground-level ozone pollution.

Landscape type is defined as a land cover mosaic composed of multiple landscape elements [15]. On the macro scale, landscape type usually refers to land cover type, while on the micro scale, it is often defined by the landscape pattern index, which represents its structural composition and spatial configuration. At present, research on the factors affecting GOCs mainly focuses on meteorological conditions [16, 17], human activities [18, 19] and social development status [20, 21]; few studies consider landscape types at different scales. With the development of remote sensing and geographic information systems, the relationship between land cover conditions and GOCs has gradually become a research hotspot. This research can be divided into two categories: one is the application of a land use regression model to predict GOCs; the other is the use of the correlation analysis method to explore the GOCs corresponding to specific land cover types [1, 2]. However, few of these studies have focused on the effects of landscape patterns on GOCs, so it is difficult to thoroughly explain the differences in the distribution characteristics of ground-level ozone pollution caused by land development activities. The prevention and control of ground-level ozone pollution requires a systematic approach that formulates countermeasures from different landscape scales. Therefore, scholars have begun to use the landscape pattern analysis method to carry out research [22, 23].

The relationship between landscape patterns and air pollutants is a complex pattern-process relationship with significant spatial heterogeneity and autocorrelation [23, 24]. Clarifying the correlation mechanism between air pollutant concentrations and landscape patterns can provide a scientific basis for controlling air pollution from the perspective of landscape pattern optimization design. The relevant research results have been applied to the landscape design of eco-friendly cities [25]. However, compared to studies related to PM_2.5_ [26], PM_10_ [27] and other atmospheric pollutants [28], few studies that consider the effect of landscape patterns on GOCs have been conducted. In addition, the spatial distribution of air quality monitoring stations in China is sparse and uneven; hence, procuring concentrations with a high spatial resolution is difficult, which is not conducive to accurately describing the relationship between landscape patterns and GOCs on the microscale.

The main objective of this paper is to analyze the variation trend in GOCs as well as their responses to landscape patterns. We first constructed a high spatial resolution concentration retrieval model to accurately describe the distribution and evolution characteristics of GOCs. Then, we calculated the landscape pattern indices over different window sizes. Pearson correlation analysis and significant test were used to quantitatively evaluate the effect of landscape pattern on the variation trend in GOCs. Finally, per-pixel rise probability of annual average GOCs at a spatial resolution of 1 km were generated.

## Material

### Study area

**Fig 1:**
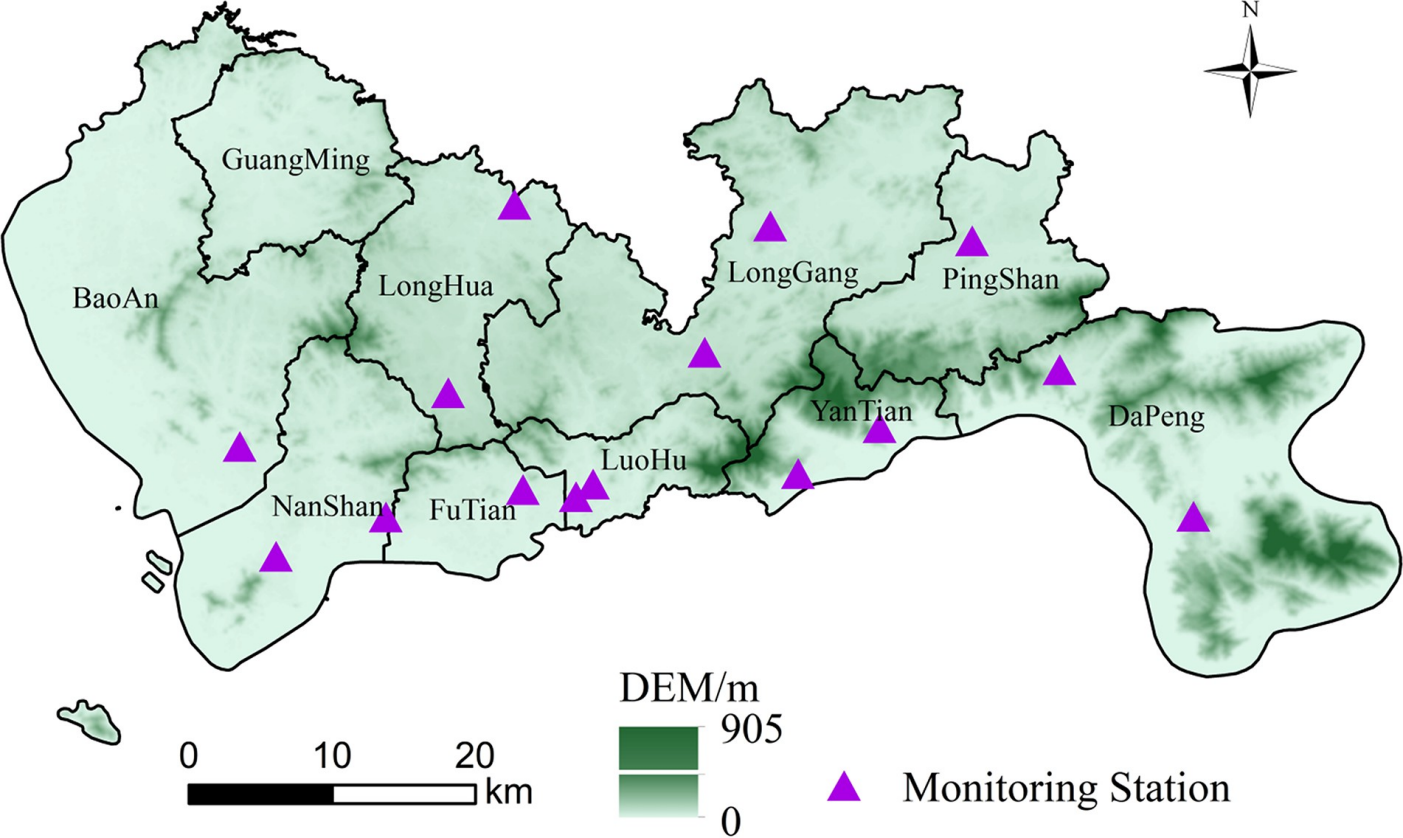
Study area and the spatial distribution of air quality monitoring stations.

Shenzhen (Fig 1), a typical area of rapid urbanization in China, was selected as the focus of this paper. It is located in the Pearl River Delta region along the southeast coast of China, in southern Guangdong Province. It lies between 113°46’~114°37’ east longitude and 22°27’~22°52’ north latitude. The total area of Shenzhen is approximately 1997.47 km^2^, and the coastline is 230 km. Shenzhen has 10 districts. They are FuTian, LuoHu, NanShan, YanTian, BaoAn, LongGang, LongHua, PingShan, GuangMing and DaPeng. The terrain is higher in the southeast and lower in the northwest.

According to the data released by Municipal Bureau of Ecology and Environment, ground-level ozone has become the primary air pollutant in Shenzhen. Shenzhen is one of the most economically developed, urbanized and densely populated cities in China. It has gradually become one of the four central cities in the Guangdong-Hong Kong-Macao Greater Bay Area. In the process of urbanization, Shenzhen has experienced severe air pollution. The unreasonable landscape configuration that characterizes the urbanization is one of the main causes. In recent years, through the adjustment of economic structure and the implementation of emission reduction measures, Shenzhen has achieved remarkable results in air pollution control. However, its ground-level ozone pollution is still serious [29]. With rapid urbanization in Shenzhen, the impact of landscape upheaval on GOCs has become prominent, which brings challenges to ground-level ozone pollution control.

### Data source and preprocessing

#### Land cover data

The land cover data set (spatial resolution: 1 km) was provided by the Data Center for Resources and Environmental Sciences, Chinese Academy of Sciences (RESDC) (http://www.resdc.cn). The data set is obtained by visual interpretation using Landsat remote sensing image data as the main information source. In Shenzhen, there are five land cover types (i.e., artificial surfaces, cultivated land, forest, grassland, and water bodies). The definition of the five land cover types is shown in Table 1.

**Table 1:** Definition of each land cover type.

Type	Definition
Artificial surfaces	Land modified by human activities, including all kinds of habitation, industrial and mining area, transportation facilities, interior urban green zones and water bodies, etc.
Cultivated land	Land used for agriculture, horticulture, and gardens, including paddy fields, irrigated and dry farmland, vegetable and fruit garden, etc.
Forest	Land covered by trees, vegetation covers over 30%, including deciduous and coniferous forests, and sparse woodland with cover 10–30%, etc.
Grassland	Land covered by natural grass with cover over 10%, etc.
Water bodies	Water bodies in land area, including river, lake, reservoir, fish pond, etc.

**Fig 2:**
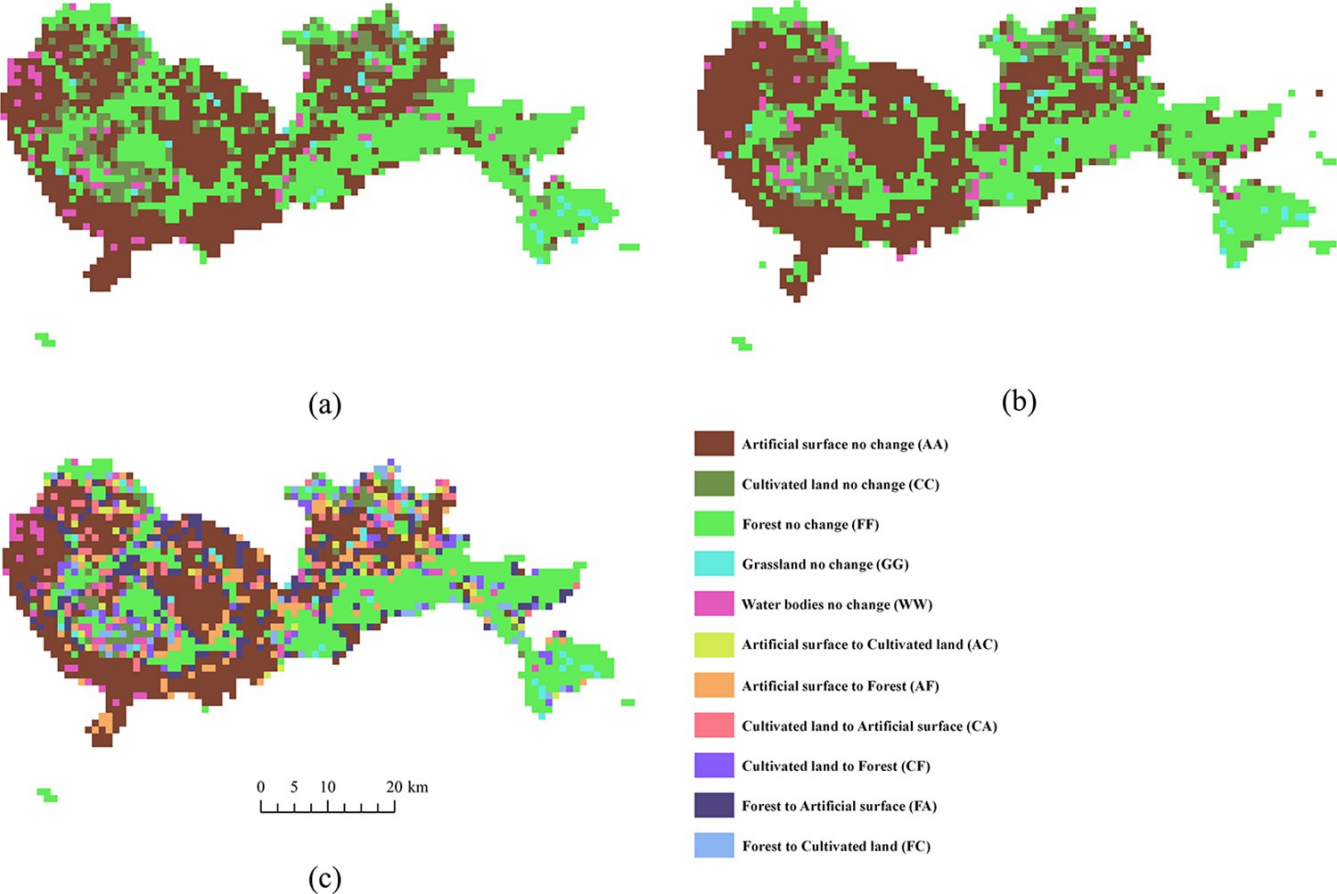
The land cover data: (a) land cover map in 2015, (b) land cover map in 2020, and (c) land cover change map from 2015 to 2020 (the contents in parentheses indicate the abbreviations for categories).

The two single-date (i.e., 2015 and 2020) land cover maps (Fig 2(A) and 2(B)) were used to create a change map with specific “from-to” change information. Theoretically, a land cover change map with 25 possible combinations of “from-to” change information could be derived from the two single-date maps. As there are some nonexistent and unlikely change categories, we kept only 11 categories in the final land cover change map, including 5 no change categories and 6 change categories, as shown in Fig 2(C).

We calculated the area proportion of different land cover change categories in each district of Shenzhen (Table 2). As described in Table 2, the main change category is FA, that is, the change from forest to artificial surface, which is closely related to the demand for construction land for rapid urbanization in Shenzhen. The land cover categories in different districts have changed to different degrees from 2015 to 2020. The highest proportion of the unchanged category is FF in DaPeng, LuoHu, PingShan and YanTian, and is AA in BaoAn, FuTian, GuangMing, LongHua, NanShan and LongGang. In the change categories, FA accounts for the highest proportion. For example, 18.50% of the area in LongHua changed from forest to artificial surface. In LongGang, NanShan, PingShan and FuTian, it is common for A change into F, accounting for 13.35%, 11.11%, 9.32% and 8.70% of each district area. For the change categories in GuangMing, CA accounts for the highest proportion, reaching 12.34% of the district area. In Dapeng, the proportion of FC and FA is the nearly same, about 5.47%.

**Table 2:** The area proportion of different change categories in each district (%).

District Categories	Da Peng	Bao An	Fu Tian	Guang Ming	Long Hua	Luo Hu	Nan Shan	Ping Shan	Yan Tian	Long Gang
A	1.56	45.18	72.46	36.36	48.55	32.47	44.44	20.50	16.67	37.06
C	0.78	4.82	0.00	3.90	0.00	0.00	6.94	1.86	0.00	4.36
F	71.09	13.86	11.59	19.48	15.61	37.66	15.97	39.13	60.00	14.44
G	3.91	0.60	0.00	1.30	1.73	1.30	0.69	1.24	3.33	2.18
W	1.17	9.04	0.00	1.95	0.58	3.90	4.17	3.11	1.67	2.45
AC	1.95	1.81	0.00	5.84	0.58	3.90	0.69	5.59	0.00	1.91
AF	2.73	3.01	8.70	5.19	6.36	5.19	11.11	9.32	1.67	13.35
CA	0.78	4.52	0.00	12.34	6.94	1.30	3.47	6.21	0.00	4.90
CF	5.08	4.22	0.00	3.25	0.58	2.60	6.94	4.35	0.00	4.36
FA	5.47	10.84	7.25	7.79	18.50	7.79	1.39	6.83	15.00	11.99
FC	5.47	2.11	0.00	2.60	0.58	3.90	4.17	1.86	1.67	3.00

#### Ground-level ozone datasets

There are 15 air quality monitoring stations distributed throughout Shenzhen (Fig 1). The daily averaged ozone concentrations for each stations were calculated by averaging hourly measurements. The daily values were estimated only if more than 75% of the hourly measurements were valid for that day [30, 31]. The hourly measurements (O3_8h) in situ observations from 13 May 2014 to 31 December 2020 were obtained from the national urban air quality real-time release platform of the China National Environmental Monitoring Center (CNEMC) (http://www.cnemc.cn/). The O3_8h represents the 8-h moving average for ozone. According to technical regulation for ambient air-quality assessment (HJ 663–2013, https://www.mee.gov.cn/), the O3_8h was valid for at least 6 of every 8h during the day. If more than two recordings were missing in an arbitrary consecutive 8-h time slot, the O3_8h data over that time slot were not calculated [10].

To accurately describe the spatiotemporal distribution characteristics and variation rules of GOCs, we collected multiple geospatial datasets (Table 3) according to predictive abilities and data accessibility for the establishment of a high spatial resolution concentration retrieval model [32]. As described in Table 3, the variables numbered 1–10 were obtained from the Modern-Era Retrospective Analysis for Research and Applications, Version 2 (MERRA-2). They were resampled to a uniform grid cell (i.e., 1 km * 1 km) by nearest-neighbor interpolation. The 11th variable (LST, spatial resolution: 1 km *1 km) was obtained from the Moderate Resolution Imaging Spectroradiometer (MODIS) product MYD11A1. The temporal range for these data is from 13 May 2014, to 31 December 2020. The longitude (LON), latitude (LAT) and day number sequence (DNS) of each grid cell were also used as candidate independent variables of the model.

**Table 3:** List of independent variables for GOCs prediction.

Number	Abbreviation	Description	Unit	Spatial resolution	Temporal resolution
1	RH	relative humidity after moisture	1	0.667° × 0.5°	Day
2	BCTP	bias corrected total precipitation	*kgm* ^−2^ *s* ^−1^	0.667° × 0.5°	Day
3	BCCMASS	black carbon column mass density	*kgm* ^−2^	0.667° × 0.5°	Day
4	OCSMASS	organic carbon surface mass concentration	*kgm* ^−2^	0.667° × 0.5°	Day
5	DUSMASS25	dust surface mass concentration-PM_2.5_	*kgm* ^−3^	0.667° × 0.5°	Day
6	SO2SMASS	SO_2_ surface mass concentration	*kgm* ^−3^	0.667° × 0.5°	Day
7	SO4SMASS	SO_4_ surface mass concentration	*kgm* ^−3^	0.667° × 0.5°	Day
8	SSSMASS25	sea salt column mass density-PM_2.5_	*kgm* ^−3^	0.667° × 0.5°	Day
9	PGENTOT	total column production of precipitation	*kgm* ^−2^ *s* ^−1^	0.667° × 0.5°	Day
10	QV2M	2-meter specific humidity	*kgkg* ^−1^	0.667° × 0.5°	Day
11	LST	land surface temperature	°*C*	1 km × 1 km	Day
12	LON	longitude	°	NA	NA
13	LAT	latitude	°	NA	NA
14	DNS	day number sequence	NA	NA	NA

## Methods

**Fig 3:**
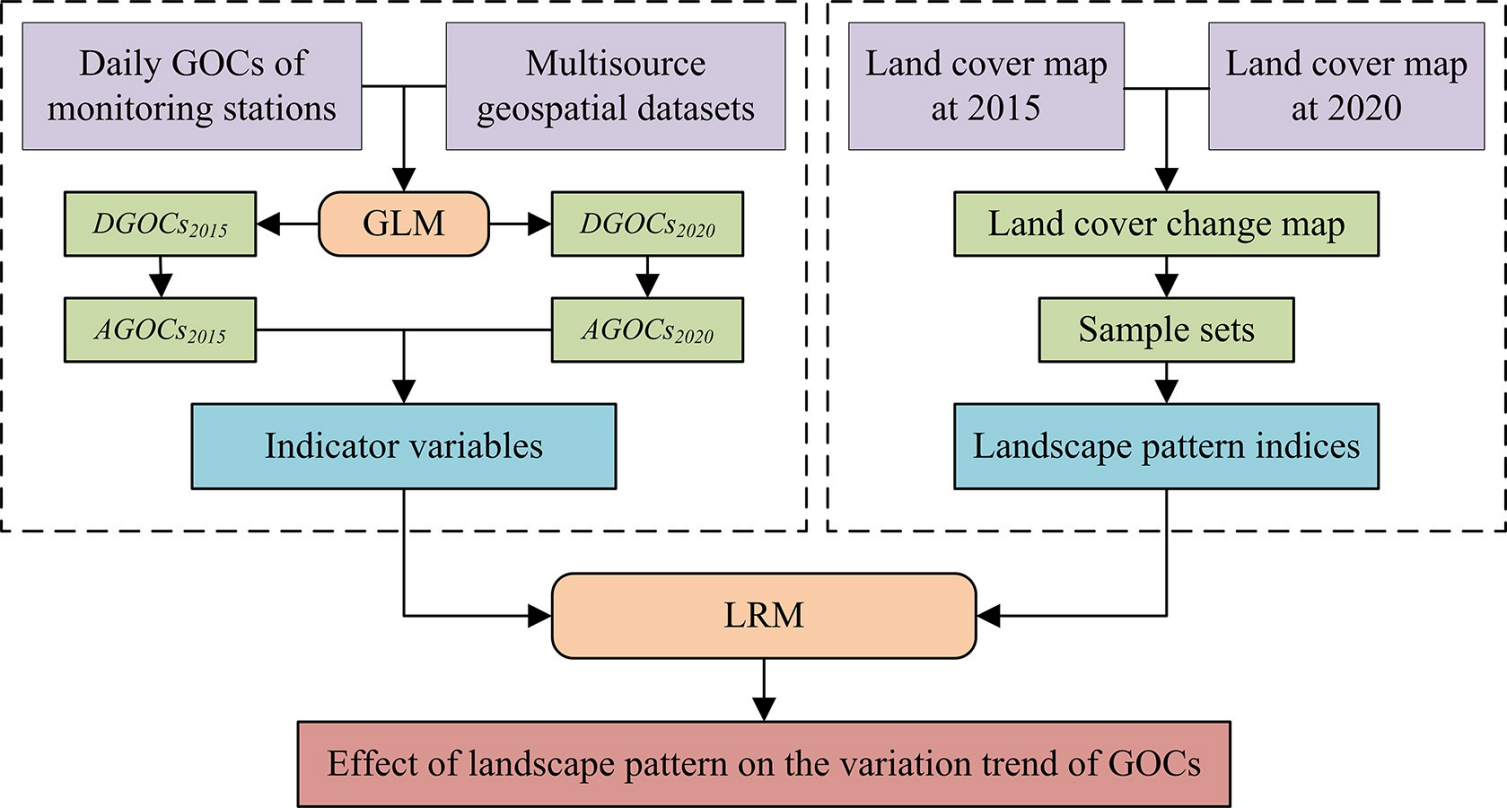
Flow chart of the hybrid method.

As described in Fig 3, a hybrid model combining a generalized linear model (GLM) and a logistic regression model (LRM) was utilized to analyze the annual variations in GOCs as well as their responses to landscape patterns in buffer zones with different radii. The per-pixel probabilities of rising concentrations at a spatial resolution of 1 km were obtained.

### GLM analysis module

The GLM analysis module was used to obtain the variation trend of annual average GOCs in Shenzhen from 2015 to 2020. First, we used a GLM to predict the daily average GOCs at a 1 km spatial resolution in 2015 and 2020 [33].

η(μ(Y))=Xβ (1)

where **X** = (*X*_0_ = 1,*X*_1_,…,*X*_*Q*_) is a vector that represents the independent variables and *Q* is the total number of independent variables, except for the constant term *X*_0_. *Y* represents the dependent variable, *η*() indicates the link function, and *η*(*Y*) represents the expected values of *Y*. **β** is the coefficient vector.

In this paper, *Y* is the daily average concentration of monitoring station *s* on day *d*, and *η*() is expressed as a log function. The multisource geospatial datasets (spatial resolution: 1 km) described in Table 3 are used as candidate independent variables. The best-fit GLM is the one that explains the greatest amount of information using the fewest possible variables. A stepwise regression method based on the Akaike Information Criterion (AIC) was utilized to identify the variables included in the final GLM [32]. The smaller the AIC value is, the better the model fit. Any variable whose *p*-value was higher than 0.05 was excluded. The daily GOCs at a 1 km spatial resolution in 2015 and 2020 (*DGOCs*_2015_ and *DGOCs*_2020_) were predicted by the best-fit GLM. Then, *DGOCs*_2015_ and *DGOCs*_2020_ were further aggregated into annual average GOCs of the corresponding year (*AGOCs*_2015_ and *AGOCs*_2020_). A 10-fold cross-validation method was performed to test the predictive ability of the GLM. The coefficient of determination (*R*^2^) and root mean square error (*RMSE*) were used as the assessment indices. An indicator variable was used to indicate whether the annual average concentrations increased (Eq (2)). According to Eq (2), *I*(*c*) is 1 if the annual average concentrations at location *c* increase from 2015 to 2020 and 0 otherwise.


$$I(c)=\{\begin{array}{cc} 1 & AGOCs{\left(c\right)}_{2015}\leq AGOCs{\left(c\right)}_{2020}\\ 0 & AGOCs{\left(c\right)}_{2015}>AGOCs{\left(c\right)}_{2020} \end{array}$$ (2)

### LRM analysis module

The LRM analysis module is used to obtain the per-pixel probabilities of rising concentrations for the land cover change maps based on the relationship between the annual average variation trend in GOCs and landscape patterns. First, we collected two sample sets from the land cover change map following the principle of stratified sampling [34], as described in Eq (3). The former set is called the training sample and the latter is the testing sample.

$$N=\frac{Z^{2}p(100-p)}{E^{2}}$$ (3)

where *p* represents the expected accuracy (in percentage form), *E* indicates the allowed error, and *Z* represents the standard normal deviation at the 95% confidence level (usually 1.96).

In this paper, we set 60% as the expected accuracy and 5% as the allowed error, resulting in 369 samples for training. In addition, 300 testing samples were collected based on the same sampling principle. The numbers of samples for individual land cover change classes are shown in Table 4. Considering the spatial resolution of the data and previous research experience [35], we established buffer zones with different radii (i.e., 3 km, 5 km and 7 km) around each sample pixel.

**Table 4:** Numbers of sample pixels for individual land cover change categories (A-artificial surfaces; C- cultivated land; F-forest; G-grassland; W-water bodies).

	Land cover change class	Number of training samples	Number of testing samples
No change category	A	124	101
	C	11	9
	F	105	85
	G	7	5
	W	12	10
Change category	A to C	8	7
	A to F	26	21
	C to A	17	13
	C to F	14	11
	F to A	35	29
	F to C	10	8

Second, we calculated the landscape pattern indices in the buffer zones with different radii for the land cover change map. To fully reflect the landscape pattern characteristics of the study area and reduce information redundancy, heterogeneity (*HET*), homogeneity (*HOM*), dominance (*DMG*) and contagion (*CON*) over different window sizes (i.e., 3*3, 5*5 and 7*7 pixels) were computed [36, 37]. The window moved from the upper left corner of the study area, one pixel at a time. The landscape pattern indices in the current window were calculated, and the results were then assigned to the central pixel of the window. *HET* refers to the number of different classes in the moving window. A heterogeneity value of 1 indicates that the central pixel is located within a homogeneous block of pixels, while any value greater than 1 indicates that the pixel is located on a patch edge or in the class boundaries. *HOM* indicates the number of pixels with the same class label as the center pixel in the moving window. *DMG* represents the deviation value between landscape diversity and maximum diversity (*H*_max_) in the moving window.

$$DMG=H_{\max}-[-\sum_{k=1}^{I}P_{k}ln(P_{k})]$$ (4)

where *P*_*k*_ is the marginal probability of individual classes *k* in the focal neighborhood and *N* is the number of land cover change classes in the region. *CON* is the extent to which different patch types are aggregated or clumped (Eq (5)).

CON=2•ln(N)−ent (5)

where $ent=-\sum_{i=1}^{N}\sum_{j=1}^{N}p_{adj}(i,j)\cdot \ln(p_{adj}(i,j))$, $p_{adj}(i,j)=N_{adj}(i,j)/(\sum_{i=1}^{N}\sum_{j=1}^{N}N_{adj}(i,j))$ and *N*_*adj*_(*i*,*j*) represent the total number of adjacencies between cells with class *i* and class *j* within a region.

Finally, the effect of landscape pattern on the variation trend of GOCs was obtained. Pearson correlation analysis and significant test was performed to explore the correlation between the landscape pattern indices and GOC variation trend. The LRM is a regression model in which the dependent variable is a binary classification variable, as described in Eq (6).

$$P=\frac{1}{\left(1+e^{-Z}\right)}$$ (6)

where $Z=\varepsilon +\sum_{i=1}^{k}b_{i}V_{i}$. As described in Eq (6), *P* represents the per-pixel probabilities of rising concentrations for land cover change maps. *V*_*i*_ (*i* = 1,⋯,*k*) indicates the explanatory variables, and *k* is the total number of explanatory variables. *ε* is a constant term. *b* represents the regression coefficient vector. The signs (positive and negative) of the regression coefficients represent the influence of explanatory variables on the probabilities of rising concentrations.

In this paper, the land cover change category (*CLA*) and the landscape pattern indices over different window sizes on the training samples were used as explanatory variables to establish the LRM. *CLA* is coded by binary variables to indicate the presence of one of the candidate land cover change categories. The indicator variable calculated based on Eq (2) was taken as the dependent variable, with a value of 1 indicating an increase in annual average concentration and a value of 0 indicating otherwise. Different explanatory variables give rise to different models, which supports the need for significance testing to select variables for regression analysis. A stepwise selection method based on the deviance statistic is used for finding the variables that have a high correlation with dependent variables (Eq (7)).

$$Deviance=-2\log\wedge =-2\sum_{l=1}^{n_{l}}\left[i_{l}\ln(\frac{{\overset{⏜}{y}}_{l}}{i_{l}})+(1-i_{l})\ln(\frac{1-{\overset{⏜}{y}}_{l}}{1-i_{l}})\right]$$ (7)

where *i*_*l*_ denotes the binomial variate for the *lth* setting of the variables, *l* = 1,⋯,*n*_*l*_ (with *n*_*l*_ being the maximum number of settings for vector **F**), and ${\overset{⏜}{y}}_{l}$ represents the model estimate corresponding to probability (*i*_*l*_ = 1|**F**_*l*_). **F** is a vector that represents the explanatory variables in the regression model. Only variables that made a significant contribution to the overall model were kept. At each step in the procedure, the significance of the addition of a variable to a model was tested. The difference in the deviance values between the two models follows a chi-square distribution with degrees of freedom (*df*), where *df* is equal to the difference between the numbers of explanatory variables in the two models [37].

To evaluate the performance of the model, we used the optimal model to predict the probability of rising concentration at the testing samples. The predicted values were transformed to indicator data at the threshold of 0.5, and the percent of samples correctly predicted (*PCP*) was calculated. The area under receiver operating characteristics curve (*AUC*) was also used as the evaluation indicator. *AUC* measures the discriminatory powers of classifiers. The *AUC* value was computed using the R package pROC, with *AUC* value closer to 1 indicating more accurate prediction [38–40].

## Results

### Ground-level ozone spatiotemporal variation characteristics

**Fig 4:**
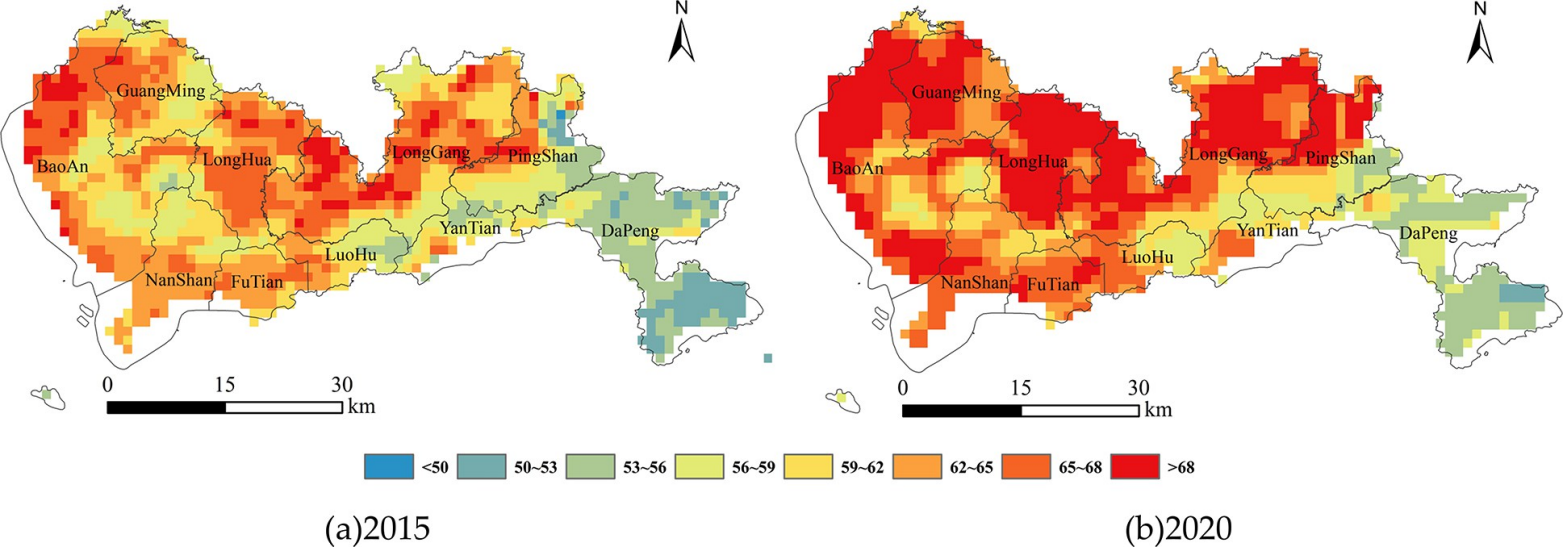
Annual average GOCs at a 1 km spatial resolution across Shenzhen in (a) 2015 and (b) 2020.

According to the AIC selection method, twelve variables (*RH*, *BCTP*, *BCCMASS*, *OCSMASS*, *DUSMASS*25, *SO*2*SMASS*, *SO*4*SMASS*, *SSSMASS*25, *LST*, *LON*, *LAT* and *DNS*) were left in the final GLM. The GLM performed satisfactorily with *R*^2^ = 0.52 and *RMSE* = 15.48%. On the basis of the daily GOCs at a 1 km spatial resolution derived from the GLM, we calculated the annual average GOCs across Shenzhen in 2015 and 2020 (Fig 4). The annual average GOCs ranged from 49.67 to 71.22 *μg*/*m*^3^ in 2015 and 52.14 to 89.89 *μg*/*m*^3^ in 2020. The two concentration maps exhibited spatial heterogeneity and an overall trend of high values in the west and low values in the east. This may be related to the more frequent air movement along the eastern seaboard. The western inland region of Shenzhen is in the pollution belt of the Pearl River Delta. Under the influence of adverse meteorological conditions such as subtropical high, the GOCs were relatively high due to the combined action of regional transport and photochemical reaction. In addition, ground-level ozone pollution is more likely to occur during the rainy season. As shown in Fig 4, the annual average GOCs in Shenzhen showed an obvious rising trend from 2015 to 2020. In 2015, the area with high GOCs was mainly concentrated in the northwest part of the city. In 2020, the GOCs rose to varying degrees in most parts of the city, including areas with high concentrations in the past. The GOCs in the northwest of Shenzhen was still high in 2020, which may be due to its relatively developed economy, industrial agglomeration, dense population, and relatively large pollutant sources.

**Fig 5:**
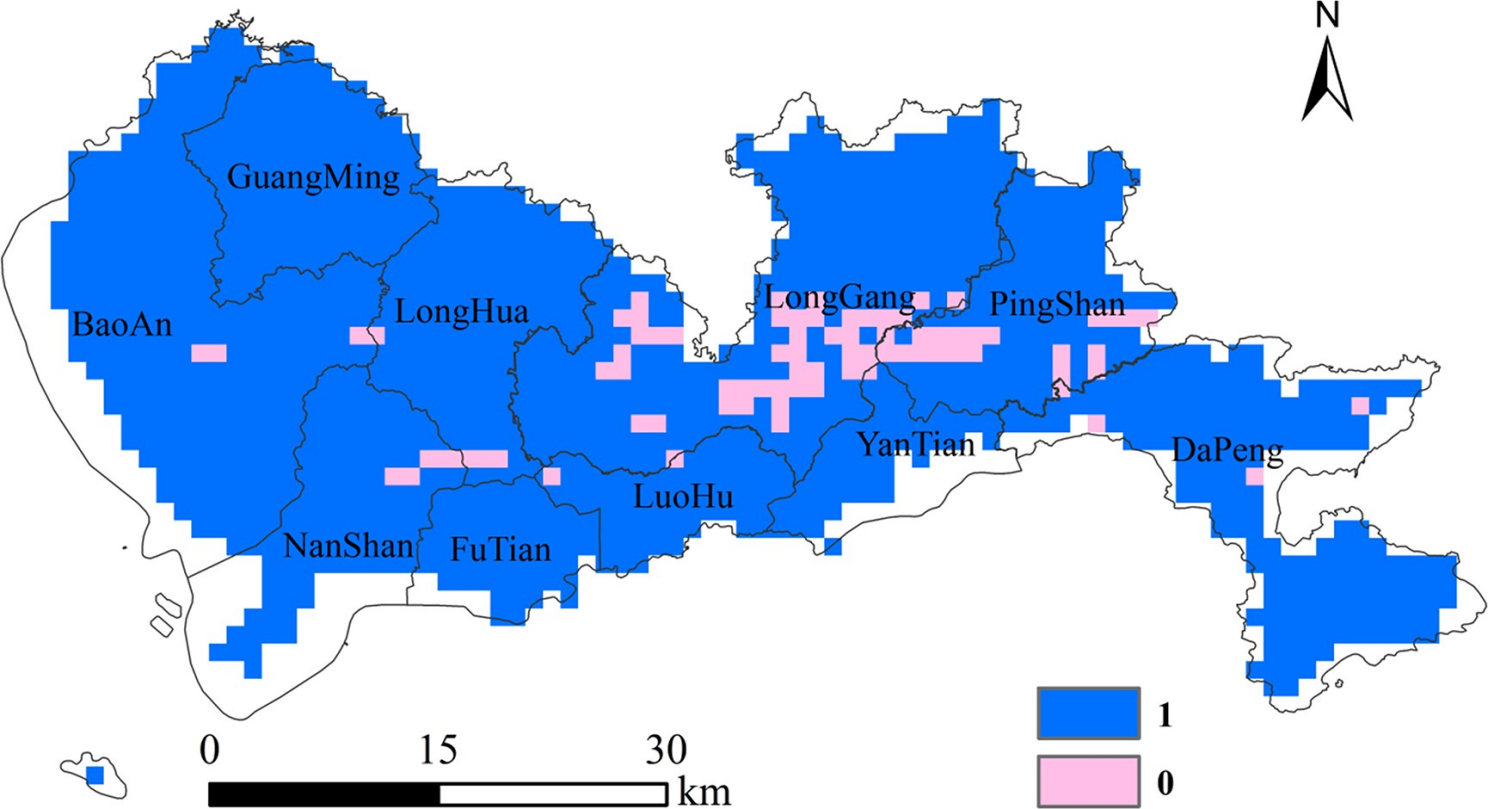
The indicator variable shows whether the annual average concentrations increased from 2015 to 2020. (1: increase, 0: not increase).

The indicator variable represented whether the annual average concentrations increased or not was obtained according to Eq (2) (Fig 5). As shown in Fig 5, the annual average GOCs in most areas of Shenzhen demonstrated an upward trend from 2015 to 2020. GOC has become a key factor restricting local air quality to reach the standard. Ground-level ozone is not directly emitted by pollutant source but is mainly generated by photochemical reactions involving ozone precursors in the presence of sunlight. Due to long-term high temperature, strong sunlight and large emission of ozone precursors, the GOCs in Shenzhen has increased significantly from 2015 to 2020. Especially in the summer with frequent typhoons, under the influence of downdraft outside the typhoon, hot and sunny weather often leads to ozone pollution in Shenzhen [41].

The areas where the concentration decreased were mainly located in LongGang and PingShan. According to the data released by Shenzhen Ecological Environment Bureau, LongGang has made great efforts to prevent and control ground-level ozone pollution. It took the lead in implementing a long-term supervision mechanism for polluting enterprises, and established a normal contact mechanism with the ecological departments of neighboring districts. Through the deployment of regional traceability monitoring points, atmospheric micro-monitoring stations and micro-atmospheric laser remote sensing radar, LongGang has realized the integration of air pollution monitoring and early warning. In addition, LongGang has fully implemented measures such as comprehensive remediation of key volatile organic compounds enterprise, low-nitrogen transformation of industrial gas boilers and road inspection of motor vehicles. PingShan has also taken a series of ground-level ozone pollution control measures. It eliminated heavily pollution enterprises and strictly controlled the construction of projects with high pollution and high energy consumption. PingShan introduced environmental quality management software to dynamically monitor air quality. In addition, it also took effective measures to increase green areas. These measures are conducive to the decrease of GOCs. But we can not ignore other factors affecting the GOCs, such as meteorological condition, human activities, economic activities and so on. Therefore, in the following study, we will explore more comprehensive and effective measures to prevent and control ground-level ozone pollution combining the impact of the above measures.

### Effect of landscape pattern on variation trend in GOCs

The relationship between landscape pattern and the variation trend in GOCs were measured according to Pearson correlation coefficient analysis and significant test (Table 5). The variation trend in GOCs is expressed by the difference between the annual average concentrations in 2020 and 2015. A correlation coefficient greater than 0 indicates a positive correlation, while a correlation coefficient less than 0 indicates a negative correlation. As shown in Table 5, the *HET* over different window sizes were significant positively correlated with the variation trend in GOCs at the significance level of 0.01, and the correlation improved with the increase of the window size. This indicates that the increase in landscape heterogeneity may increase the probability of rising concentrations, and the enhancement effect is more obvious in a wider range. There was a negative correlation between the *HOM* over different sizes and the variation trend, but not significantly. The *DMG* and *CON* over different window sizes were negatively correlated with the variation trend, and the correlation improved with the increase of window sizes. The *DMG* is mainly used to determine the dominance degree of patch types in the landscape. The index is approximately inversely proportional to the landscape diversity index. For areas with the same number of landscape types, the greater the diversity value and the smaller the dominance index, indicating that the more dispersed the land use structure. According to the correlation analysis and significant test, the higher the land use structure monopoly, the more obvious the inhibition effect on the probability of rising concentrations. The *CON* describes the degree of aggregation or extension of different patch types. A smaller value of *CON* indicates that there are many small patches in the landscape, and the overall landscape is more fragmented. The correlation analysis and significant test results showed that the more fragmented the landscape, the greater the probability of rising concentrations, especially in a wider range.

**Table 5:** Correlation coefficients between landscape pattern indices and the variation trend in GOCs (numbers after landscape pattern indices are window size).

Landscape pattern index	Correlation coefficient
*HET*3	0.13***
*HOM*3	-0.02
*DMG*3	-0.09*
*CON*3	-0.09*
*HET*5	0.26***
*HOM*5	-0.02
*DMG*5	-0.21***
*CON*5	-0.20***
*HET*7	0.30***
*HOM*7	-0.01
*DMG*7	-0.27***
*CON*7	-0.27***

***、** and *: significate at levels of 0.01, 0.05 and 0.1, respectively; sample size:369.

### Prediction of the per-pixel probability of rise concentrations

The LRM was utilized for the prediction of per-pixel probabilities of rise concentrations for the land cover change map. Several landscape pattern indices in the buffer zones with different radii were used as explanatory variables in the regression model. The exhaustive model selection procedure described in Methods section was applied to identify the model containing the highest number of significant explanatory variables (at *α* = 0.05) (Table 6). The numbers after the explanatory variables (except for *CLA*) shown in Table 6 are the sizes of the moving windows surrounding a sample pixel. At each step in the procedure, the significance of the addition of an explanatory variable (*V*_*a*_) to a model already containing variables *V*_*s*_ was tested.

**Table 6:** Chi-square tests for selected models.

Chi-square test (*df*)	Significance of an additional variable *V*_*a*_ to a model already containing variables *V*_*s*_		Difference in chi-square values	Significance at *α* = 0.05
	*V*_*s*_	*V*_*a*_		
D0-D1a (1)	1	*CLA*	21.35	Yes
D1a-D2a (1)	1,*CLA*	*HET*3	4.37	Yes
D1a-D2b (1)	1,*CLA*	*HOM*3	0.11	No
D1a-D2c (1)	1,*CLA*	*DMG*3	1.56	No
D1a-D2d (1)	1,*CLA*	*CON*3	1.37	No
D1a-D2e (1)	1,*CLA*	*HET*5	22.64	Yes
D1a-D2f (1)	1,*CLA*	*HOM*5	0.17	No
D1a-D2g (1)	1,*CLA*	*DMG*5	13.53	Yes
D1a-D2h (1)	1,*CLA*	*CON*5	12.84	Yes
D1a-D2i (1)	1,*CLA*	*HET*7	28.31	Yes
D1a-D2j (1)	1,*CLA*	*HOM*7	0.79	No
D1a-D2k (1)	1,*CLA*	*DMG*7	25.50	Yes
D1a-D2l (1)	1,*CLA*	*CON*7	25.96	Yes
D2i-D3a (1)	1,*CLA*,*HET*7	*HOM*7	5.37	Yes
D2i-D3b (1)	1,*CLA*,*HET*7	*DMG*7	0.74	No
D2i-D3c (1)	1,*CLA*,*HET*7	*CON*7	1.33	No
D3a-D4a (1)	1,*CLA*,*HET*7,*HOM*7	*DMG*7	7.94	Yes
D3a-D4b (1)	1,*CLA*,*HET*7,*HOM*7	*CON*7	9.18	Yes
D4b-D5a (1)	1,*CLA*,*HET*7,*HOM*7,*CON*7	*DMG*7	1.78	No

**Fig 6:**
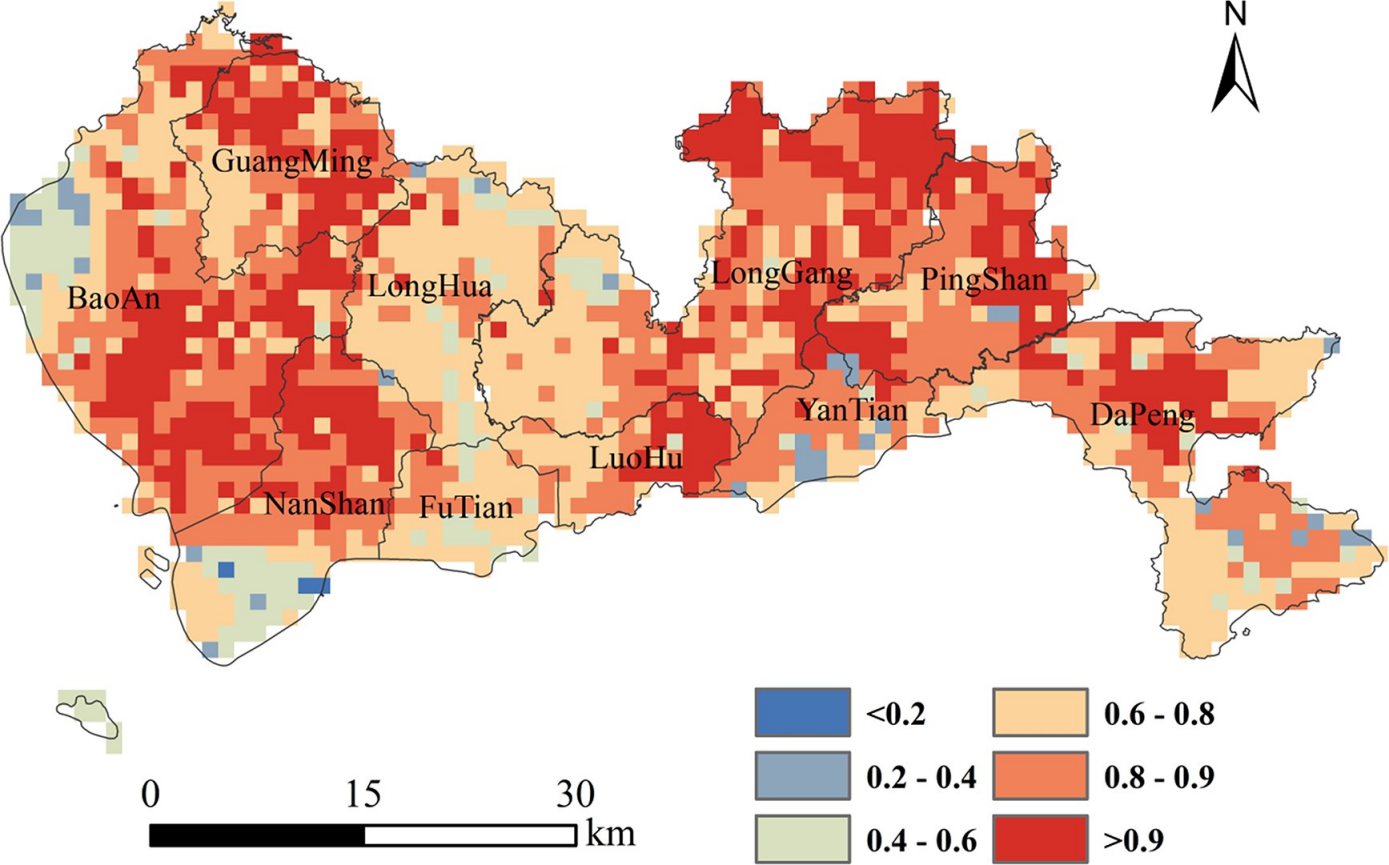
Per-pixel probability map of rising concentrations in Shenzhen from 2015 to 2020.

As shown in Table 6, most of the explanatory variables were significant if the model contained *CLA*, except for *HOM*3, *DMG*3, *CON*3, *HOM*5 and *HOM*7. The landscape pattern indices over a window size of 7*7 pixels were more significant than others, with *HET*7 being the most significant if *CLA* were already in the model (Model 2i). Therefore, the analysis was continued with Model 2i (*CLA* and *HET*7). Adding *DMG*7 or *CON*7 to Model 2i was not significant, whereas adding *HOM*7 to Model 2i was significant. Adding *CON*7 to Model 3a (*CLA*, *HET*7 and *HOM*7) was more significant. Finally, it was proven that adding *DMG*7 to Model 4b (*CLA*, *HET*7, *HOM*7 and *CON*7) would not significantly improve the fit. Thus, Model 4b (*CLA*, *HET*7, *HOM*7 and *CON*7) contained the highest number of significant variables. The model exhibited satisfactory performance (*PCP* = 82.33%, *AUC* = 0.70). The per-pixel probability of rise concentrations in Shenzhen during 2015–2020 were generated from the model (Fig 6).

**Fig 7:**
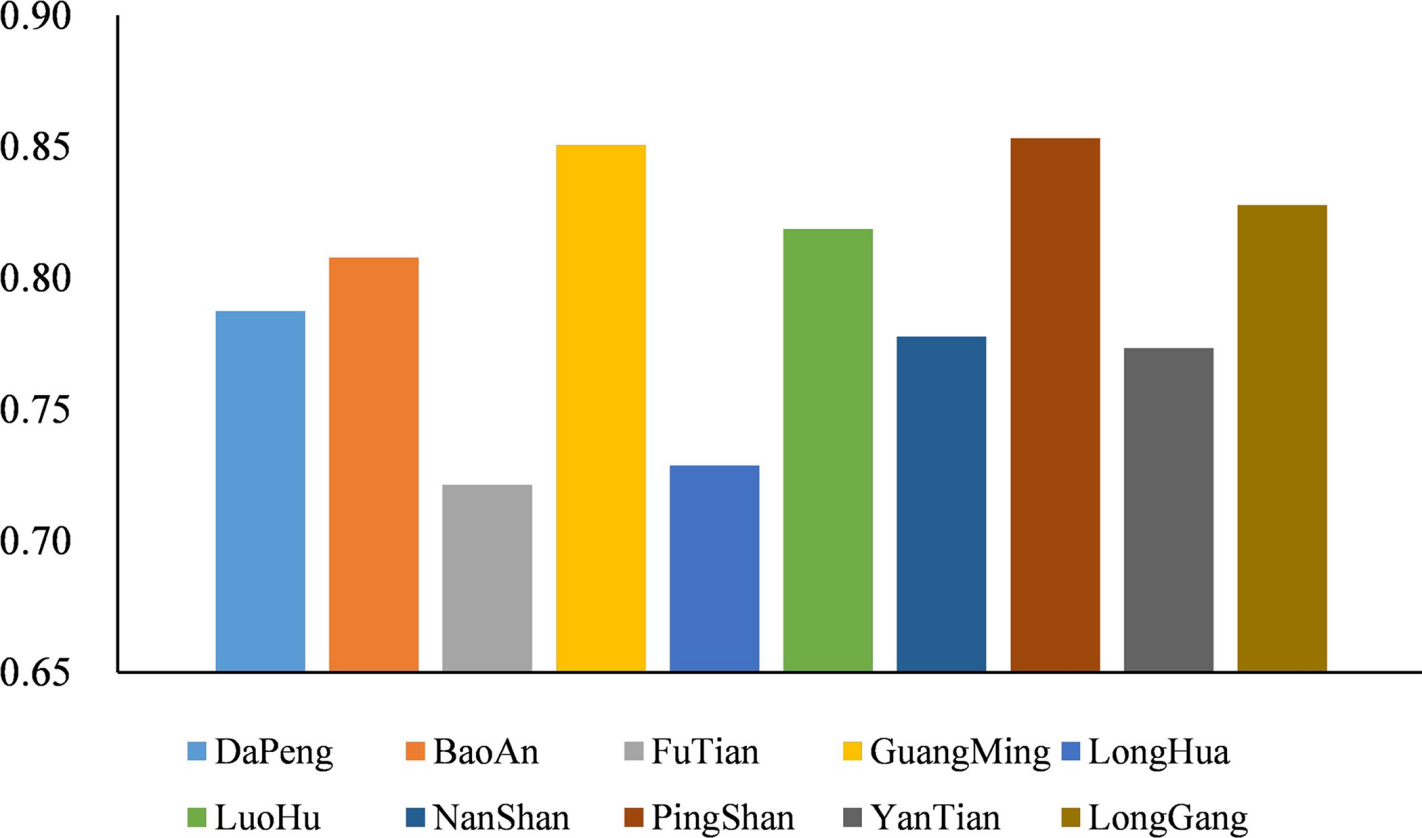
Mean value of the rise probability in each district of Shenzhen.

In recent years, through the adjustment of economic structure and the implementation of emission reduction measures, Shenzhen’s air quality has improved significantly. However, its ground-level ozone pollution is increasingly prominent, and there is a trend of worsening pollution risk. As shown in Fig 6, compared with 2015, the rise probability of annual average GOCs in most areas in 2020 was bigger than 0.6, indicating that the GOCs in Shenzhen still has a great risk of rising. We calculated the mean of the rise probability in each district of Shenzhen (Fig 7). As shown in Fig 7, there is spatial heterogeneity in the distribution of the rise probability of average annual GOCs in Shenzhen. The mean values of each district are above 0.70, indicating a high likelihood of rising concentrations in these areas. The districts (i.e., GuangMing, PingShan, LongGang, LuoHu and BaoAn) have higher mean values, which should be paid more attention in the prevention and control of ground-level ozone pollution. Combined with the effect of landscape pattern on the variation trend in GOCs, the rise probability of average annual GOCs can be suppressed by reducing landscape heterogeneity or improving landscape dominance and aggregation. It will contribute to the synergistically promoting of ground-level ozone pollution control and landscape optimization design. Even so, it cannot be ruled out that local meteorological conditions, human activities and land cover condition contribute to the variation trend in GOCs, which requires further study.

## Discussion

The accelerated development of urbanization has brought about a series of problems, such as land shortages, population surges, and traffic congestion, which have led to severe air pollution. Landscape type has an important effect on the urban atmospheric environment. In the limited urban development space, optimizing the landscape type plays an important role in improving the quality of the atmospheric environment. As the largest developing country, China’s urbanization rate continues to increase. By 2022, China’s urbanization rate reached 65.22%. With the rapid growth of urbanization, the contradiction between land resource supply and demand has become increasingly prominent. The problem of ground-level ozone pollution caused by unreasonable landscape planning has gradually become an important factor restricting the sustainable development of society in China. According to the air quality report released by the Ministry of Ecology and Environment of the People’s Republic of China, ground-level ozone has become the primary pollutant in many urban areas of China. Urbanization is the inexorable trend of social development. In the process, landscape changes are inevitable. Exploring the relationship between landscape types and GOCs can guide landscape planning in controlling ground-level ozone pollution, so as to realize the coordinated promotion of atmospheric environmental protection and social development.

Literature research shows that most studies on the relationship between landscape types and GOCs describe the concentrations corresponding to specific land cover types on the macro scale. Few studies examine the influence of landscape patterns on GOCs at the microscale, and it is difficult to explain the differences in GOCs caused by landscape structure composition and spatial configuration. Therefore, the relationship between landscape patterns and GOCs has become a hot issue in the field of ground-level ozone pollution control. However, this type of study is faced with problems such as the limited spatial data of ground-level ozone, complex processes and mechanisms of landscape patterns affecting GOCs, and uncertainty of microscale modeling.

In view of the urgent need for mitigating ground-level ozone pollution through rationally planned multiscale landscapes, this study addressed the variation trend prediction of GOCs at a high-spatial resolution as well as their relationship with landscape patterns. First, a high spatial resolution concentration retrieval model was established based on multisource geospatial data and in situ ground-level ozone data. The derived GOCs alleviated the restriction of ground-level ozone spatial data quality and provided data support for the quantitative analysis of the relationship between landscape patterns and the variation trend in GOCs. Second, specific landscape elements and spatial characteristics were quantified by using landscape pattern indices over different window sizes. Then, the internal information and composition of land cover change information were explained. The calculation of these indices is relatively quick and direct, which is convenient for exploring the influence of landscape pattern characteristics on concentration variation trends. Third, the per-pixel probability of rise concentrations at a spatial resolution of 1 km was derived. The results help to better understand the distribution of ground-level ozone pollution and provides scientific basis for controlling ground-level ozone pollution from the perspective of landscape planning.

The main limitation of the study is that the mechanism and process of landscape pattern affecting the variation trend in GOCs had not been fully explored, which needs further study. Variation trend in GOCs was influenced by many factors besides the variables in our study, and this would definitely be in our further research. It is difficult to investigate the effects of multi-scale landscape types on GOCs due to the lack of ground-level ozone data. Therefore, we will continue to investigate concentration retrieval models to provide spatially continuous distribution and long-term change characteristics for GOCs. In addition, the regional and seasonal difference of the effects, the contribution degree of “source” and “sink” landscapes and their driving factors would also be the focus of future study.

## Conclusions

To analyze the variation trend in GOCs as well as their response to landscape patterns, a hybrid model combining a GLM and a LRM was developed. The hybrid model exhibited satisfactory performance (*PCP* = 82.33% and *AUC* = 0.70) in predicting per-pixel rising probabilities of annual average GOCs at a spatial resolution of 1 km. Pearson correlation analysis and significance test were utilized in analyzing the relationship between landscape patterns and variation trend in GOCs. The study yielded the following findings:

1. The annual average GOCs in Shenzhen had spatial heterogeneity and showed an obvious upward trend during 2015–2020. The GOCs tend to form and accumulate to higher levels in the western region, especially in the northwest near the inland region.
2. The landscape pattern indices (i.e., *HET*, *DMG*, *CON*) over different window sizes were significantly related with the variation trend in GOCs. The landscape with low value of *HET* and high values of *DMG* and *CON* may inhibit the rise of GOCs.
3. The predicted rise probability of annual average GOCs revealed spatiotemporal variation characteristics which are valuable for ground-level ozone pollution control and prevention. The GOCs in Shenzhen still has a great risk of rising, especially in GuangMing, PingShan, LongGang, LuoHu and BaoAn.

The results provide preliminary indicators for estimating the variation trend of GOCs in the absence of monitoring data and improves the reliability of alleviating ground-level ozone pollution by optimizing landscape design.
